# Supplementary material for: Beyond the leaderboard: leveraging predictive modeling for protein–ligand insights and discovery
Source: Bioinformatics. 2025 Aug 7;41(8):btaf425. doi: 10.1093/bioinformatics/btaf425 (PMC12342388; doi:10.1093/bioinformatics/btaf425)
Supplement: btaf425_Supplementary_Data [file btaf425_supplementary_data.pdf]

S1 ProtLigand Algorithm

We introduce **ProtLigand**, a novel ligand-aware PLM that enhances protein representations by leveraging information from interacting ligands.

Let a protein $p = (S, R)$ be defined by its amino acid sequence $S = (s_1, s_2, \ldots, s_n)$ and its 3D structure R, where $s_i \in \mathcal{V}$ denotes the i-th residue and $\mathcal{V}$ is the standard residue alphabet. We then use Foldseek (van Kempen *et al.*, 2022) to convert each protein structure into a sequence of 3Di tokens $F = (f_1, f_2, \ldots, f_n)$ aligned with S, where each structural token $f_i \in \mathcal{F}$ from a structure alphabet $\mathcal{F}$. Then, we construct a structure-aware sequence $P = (s_1f_1, s_2f_2, \ldots, s_nf_n)$, where $s_if_i \in \mathcal{V} \times \mathcal{F}$ token combines residue identity and local structural context. This fused sequence can then be fed into a standard Transformer encoder as basic input.

S1.0.1 Training Phase

During training, each structure-aware protein sequence P is passed through a protein encoder, producing a contextual representation $z_p \in \mathbb{R}^{N \times D}$, where N is the sequence length and D is the feature dimension.

We initialize the encoder with a large pre-trained PLM, SaProt-650M (Su *et al.*, 2024), leveraging its strong contextual understanding of structure-aware sequences. Using SaProt allows us to fairly isolate and assess the contribution of ligand integration. Alternatively, other advanced PLMs can also be used within the proposed framework.

For each protein, we retrieve its known interacting ligand from our pre-training dataset (see Section 2.1). The ligand is represented as a SMILES string $L = (l_1, l_2, \ldots, l_M)$ and passed through a pre-trained molecular encoder (ChemBERTa-77M-MLM (Chithrananda *et al.*, 2020)), producing a chemically meaningful ligand representation $z_l \in \mathbb{R}^{M \times D_L}$, where M is the ligand sequence length and D_L is its feature dimension.

To align the ligand and protein representations in a shared latent space, ProtLigand applies an affine transformation to map the ligand representation to the protein space:

$$z'_l = \text{Linear}(z_l) \in \mathbb{R}^{M \times D}$$

Next, we apply cross-attention (Vaswani *et al.*, 2017) between the protein and ligand tokens. The ligand-derived keys and values guide the protein's queries, refining its representation to reflect ligand-binding context. The attention mechanism is defined as:

$$Q = z_p W_Q$$
$$K = z'_l W_K$$
$$V = z'_l W_V$$
$$z'_p = \text{softmax}\left(\frac{QK^\top}{\sqrt{d}}\right) V$$

Here, $W_Q, W_K, W_V \in \mathbb{R}^{D \times D}$ are learnable projection matrices. The resulting $z'_p \in \mathbb{R}^{N \times D}$ is a ligand-aware protein representation that captures the biochemical influence of ligand interactions.

S1.0.2 Masked Language Modeling Training Objective

For each input protein, 15% of the amino acids are randomly masked. Each masked amino acid $s_{(i)}$ has an 80% chance of being masked for prediction, 10% chance of replacement with a random amino acid, and 10% chance of remaining unchanged. Suppose the number of masked amino acids is N, the training objective $\mathcal{L}_{MLM}$ is to minimize:

$$\min_\theta \mathcal{L}_{MLM} = -\sum_{i=1}^{N} \log P(s_{(i)} | z'_p, \Theta) \quad \text{(S1)}$$

where z'_p is the ligand-aware protein representation and Θ are the parameters of the ProtLigand model.

S1.0.3 Ligand Generator

During inference, experimentally determined ligand information is often unavailable. To eliminate this dependency, we introduce a ligand generator module trained separately during the pre-training phase. This component learns to predict a proxy representation of the interacting ligand directly from the protein representation.

The ligand generator is implemented as a Transformer encoder with L_1 layers and H_1 heads each, followed by a linear projection. Given the protein representation $z_p \in \mathbb{R}^{N \times D}$ produced by the protein encoder, the generator computes:

$$\hat{z}_p = \text{TransformerEncoder}(z_p) \in \mathbb{R}^{N \times D}$$
$$\hat{z}_l = \text{Linear}(\hat{z}_p) \in \mathbb{R}^{N \times D_L}$$

where D_L is the ligand representation dimension, aligned with the output of the pre-trained ligand encoder. To assess the quality of the generated ligand representations, we minimize the cosine distance between the predicted ligand representation $\hat{z}_l$ and the ground-truth ligand representation derived from the ligand encoder z_l:

$$\mathcal{L}_{\text{rec}} = 1 - \frac{\hat{z}_l \cdot z_l}{\|\hat{z}_l\| \|z_l\|} \quad \text{(S2)}$$

This loss encourages the protein encoder to capture ligand-relevant features, allowing ProtLigand to operate effectively even in the absence of known ligand data at inference time.

S1.0.4 Ligand Decoder

The ligand decoder is trained to reconstruct the SMILES sequence from the generated ligand representation $\hat{z}_l \in \mathbb{R}^{N \times D_L}$. It consists of an embedding layer, learnable positional encoding, a Transformer decoder with L_2 layers and H_2 heads each, and a linear output layer with output dimension equal to the vocabulary size.

Given a target ligand sequence t, the decoder computes:

$$e_t = \text{Embedding}(t) + \text{PositionalEncoding}(t)$$
$$\text{logits} = \text{TransformerDecoder}(e_t, \hat{z}_l)$$
$$\hat{t} = \text{Linear}(\text{logits})$$

To assess the quality of the SMILES reconstruction, we minimize the cross-entropy loss between the predicted token distribution and the ground-truth SMILES sequence:

$$\mathcal{L}_{\text{SMILES}} = \text{CrossEntropy}(\hat{t}, t) \quad \text{(S3)}$$

We trained the ligand decoder alongside the ligand generator, minimizing the mutual loss $\mathcal{L} = \mathcal{L}_{\text{SMILES}} + \mathcal{L}_{\text{rec}}$.

Table S1. Training hyperparameters for ProtLigand and its ligand generator.

Parameter	ProtLigand Pre-training	Ligand Generator Pre-training	Fine-tuning (Inference)
Optimizer	AdamW	AdamW	AdamW
β_1, β_2	0.9, 0.98	0.9, 0.98	0.9, 0.98
Weight Decay	0.01	0.01	0.01
LR Init / Peak / Final	2e-5 / 1e-4 / 5e-5	6e-5	2e-5
LR Schedule	Warmup + Linear Decay	Fixed	Fixed
Warmup Steps	10K	–	–
Decay Start / End	30K / 70K	–	–
Training Epochs	100	100	Task-specific
Batch Size	16	16	64

S1.0.5 Inference Phase

During inference, we leverage the ligand generator (see Supplementary Section S1.0.3) to produce the interacting ligand representation from the protein representation, eliminating the need for known ligand input. Then, given the ligand representation and the protein, ProtLigand produces the ligand-aware protein representation. Furthermore, task-specific classification heads are incorporated to enable predictions for the specific downstream tasks. This allows the model to remain ligand-aware without external annotations and generalize to unseen proteins.

We present an example of the inference phase for the HumanPPI task in Supplementary Figure S1, where the model predicts whether two given proteins interact.

To decode the interacting ligand representations into SMILES sequences, we generate molecules autoregressively using top-k sampling ($k = 3$), until a termination token is produced. The chemical validity of each generated ligand is verified using RDKit. Representative examples of valid generations are shown in Figure 1.

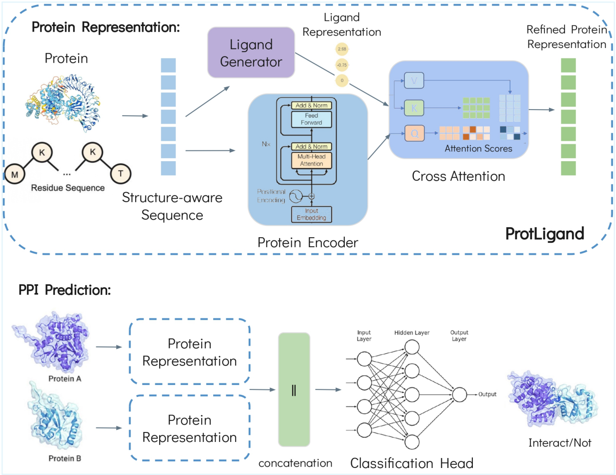

Fig. S1. Illustration of the inference pipeline for the HumanPPI classification task.

S1.1 Baselines

Incorporating SOTA models as baselines and following SaProt (Su *et al.*, 2024) for fair comparison, we include ESM-1b (Rives *et al.*, 2019) and ESM-2 (Lin *et al.*, 2023), which are considered top-performing sequence-based models. Structure-based baselines include GearNet (Zhang *et al.*, 2023a) and MIF-ST (Yang *et al.*, 2022), while ESM-GearNet (Zhang *et al.*, 2023b) serves as a representative joint sequence–structure model. We also evaluate against SaProt itself (Su *et al.*, 2024), the current leading PLM that incorporates AlphaFold-derived structure tokens.

S1.2 Tasks

We provide a comprehensive overview of the downstream tasks, selected following the latest SOTA SaProt (Su *et al.*, 2024), to rigorously evaluate the effectiveness of our proposed approach for protein representation learning.

S1.2.1 Protein-Protein Interaction Prediction

Reliable detection of protein–protein interactions (PPIs) is essential for deciphering cellular processes and uncovering therapeutic targets, especially when the interactions have not been previously characterized (Humphreys *et al.*, 2021). We use the HumanPPI dataset from the PEER benchmark (Xu *et al.*, 2022) to evaluate binary interaction prediction between protein pairs.

Following established best practices in this task (Xu *et al.*, 2022; Su *et al.*, 2024), we report accuracy as the primary evaluation metric. In addition, we include AUROC (Area Under the Receiver Operating Characteristic Curve), which is widely used in the biomedical literature (Capel *et al.*, 2022; Shin *et al.*, 2021) for its ability to reflect classification performance across thresholds.

S1.2.2 Protein Function Prediction

We evaluated protein function using two tasks: thermostability prediction on the “human-cell” split of the FLIP benchmark (Dallago *et al.*, 2021), formulated as a regression task and evaluated using Spearman’s rank correlation coefficient (ρ); and Metal Ion Binding (Hu *et al.*, 2022), a binary classification task designed to predict the presence of metal ion binding sites within a protein, evaluated by accuracy. Besides the best practices in this task (Dallago *et al.*, 2021; Su *et al.*, 2024) for application usages, we report AUROC, which is common in these tasks (Capel *et al.*, 2022; Shin *et al.*, 2021).

S1.2.3 Protein Localization Prediction

We adopt the DeepLoc dataset (Armenteros, 2017), which provides two variants of the subcellular localization task: (1) a binary classification task and (2) a 10-class multiclass classification task. We use accuracy as the primary performance metric for both variants, and additionally report AUROC for the binary classification task, following similar studies in this field (Capel *et al.*, 2022; Shin *et al.*, 2021).

S1.2.4 Protein Annotation Prediction

We evaluate protein functional annotation using the Enzyme Commission (EC) number prediction task from the DeepFRI

benchmark (Gligorijević *et al.*, 2021). This task is formulated as a multi-label classification problem, where each protein may be assigned one or more EC labels. $F_{\max}$ score is used for evaluation.

S1.3 Implementation Details

S1.3.1 Pre-Training Phase

We utilized the pre-trained SaProt 650M (Su *et al.*, 2024) as the base protein encoder and ChemBERTa-77M-MLM (Chithrananda *et al.*, 2020) as the base ligand encoder. Following ESM-2 (Lin *et al.*, 2023) and BERT (Devlin *et al.*, 2019), 15% of the tokens are randomly masked during training.

The hyperparameters of both ProtLigand and the ligand generator (summarized in Supplementary Table S1) were selected via grid search based on performance on the validation set of the pre-training dataset. As a result, the encoder and decoder are configured with $L_1 = L_2 = 6$ layers and $H_1 = H_2 = 8$ attention heads per layer. The dimensionalities of the protein and ligand representations are set to $D = 1280$ and $D_L = 768$, respectively. To accommodate long protein sequences, inputs are truncated to a maximum of 1,024 tokens. All training is performed using mixed-precision arithmetic to improve memory efficiency and computational throughput.

S1.3.2 Fine-Tuning Phase

During inference, we incorporate task-specific classification heads to enable predictions for these tasks. Furthermore, following SaProt (Su *et al.*, 2024) settings, we conducted evaluations of our model and all baselines using the same set of hyperparameters reported in SaProt to ensure fair comparisons. These hyperparameters are summarized in Supplementary Table S1.

S1.3.3 Computational Complexity

All experiments were conducted on 4× NVIDIA A100 80GB GPUs. ProtLigand's pre-training required approximately 72 hours (288 GPU hours), with most overhead stemming from its ligand generator and cross-attention modules. In contrast, SaProt (Su *et al.*, 2024) was trained from scratch over three months using 64 GPUs. Thus, ProtLigand achieves a 99% reduction in pre-training cost by leveraging a pretrained encoder and integrating ligand context through lightweight modules.

At inference time, ProtLigand introduces only modest overhead. SaProt inference takes approximately 0.014 seconds per 1,000 residues, whereas ProtLigand processes the same input in about 0.017 seconds—representing a 21% increase. This overhead is minimal given the improved predictive performance and full reuse of pre-trained PLM infrastructure.

S2 Statistical and Reliability Evaluation

S2.1 Statistical Significance Tests

We perform paired two-tailed t-tests to assess the statistical significance of ProtLigand's improvements over the top-performing baseline, SaProt (Su *et al.*, 2024), across all six benchmark tasks. Holm–Bonferroni correction is applied across 3 runs; the maximum adjusted p-value is reported per task. The raw and adjusted p-values are summarized in Supplementary Table S2. Statistically significant differences ($p < 0.05$) indicate that the observed performance improvements are unlikely to have occurred due to stochastic variation.

Table S2. Raw and Holm–Bonferroni-adjusted p-values for ProtLigand and SaProt across all tasks. Raw p-values are computed using a paired two-tailed t-test over per-sample prediction scores across 3 fine-tuning runs.

Task	Raw p-values	Adjusted p-value
HumanPPI	0.0120, 0.0140, 0.0130	0.0360
Thermostability	0.0003, 0.0001, 0.0002	0.0004
Metal Ion Binding	0.0150, 0.0170, 0.0160	0.0450
DeepLoc Binary	0.0090, 0.0110, 0.0100	0.0270
DeepLoc Sub.	0.0120, 0.0100, 0.0110	0.0300
EC	0.0070, 0.0060, 0.0050	0.0210

S2.2 Confidence Intervals

To provide a clearer estimate of uncertainty across the three independent fine-tuning runs, we report 95% confidence intervals (CIs) for ProtLigand alongside the mean and standard deviation (Supplementary Table S3). Confidence intervals are computed using a two-tailed t-distribution with $n = 3$.

Table S3. Mean ± standard deviation and 95% confidence intervals for ProtLigand across the six benchmark tasks.

Task	Mean ± SD	95% CI
HumanPPI (Acc%)	90.000 ± 0.5	[88.76, 91.24]
Thermostability (ρ)	0.731 ± 0.02	[0.680, 0.780]
Metal Ion Binding (Acc%)	77.54 ± 0.18	[77.09, 77.99]
DeepLoc Binary (Acc%)	94.02 ± 0.21	[93.50, 94.54]
DeepLoc Sub. (Acc%)	83.88 ± 0.07	[83.71, 84.05]
EC ($F_{\max}$)	0.887 ± 0.002	[0.880, 0.890]

S2.3 AUROC Evaluation for Classification Tasks

To complement the accuracy-based evaluation, we report the AUROC scores for binary classification tasks (HumanPPI, Metal Ion Binding, and DeepLoc Binary) in the Supplementary Table S4. AUROC provides a threshold-independent measure of model performance, which is more rigorous in certain tasks. ProtLigand consistently achieves the highest AUROC in all evaluated tasks, compared to the SOTA model in each category, reflecting a trend similar to the accuracy results reported and demonstrating its robust and reliable classification capability.

Table S4. AUROC scores for the binary classification tasks. Statistically significant results with $p < 0.05$ using a two-tailed paired t-test are marked with an asterisk (*). For ProtLigand, we report the mean and standard deviation over 3 independent fine-tuning runs (with different seeds). The best result is highlighted in bold.

Model	HumanPPI	Metal Ion.	DeepLoc
ESM-2	0.748	0.786	0.951
SaProt	0.926	0.832	0.958
**ProtLigand**	**0.957***±0.02	**0.849***±0.002	**0.968***±0.001

S2.4 Ligand Context Boosts Prediction Confidence

The performance gap observed in downstream tasks, such as HumanPPI, reflects only the difference in success rates, in cases where ProtLigand correctly predicts interactions and SaProt does not. However, another crucial aspect highlighted in Supplementary Figure S3 is ProtLigand's consistently higher

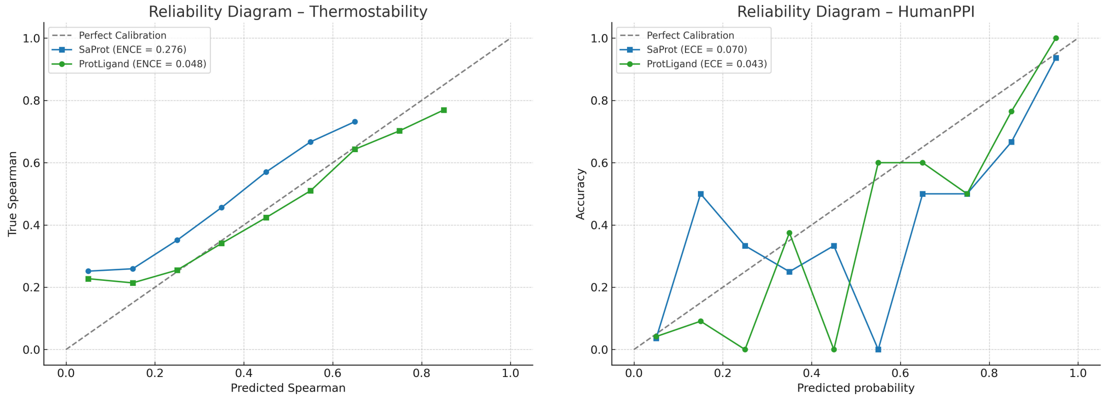

(a) Thermostability (Regression) (b) HumanPPI (Classification)

Fig. S2. Reliability diagrams for Thermostability and HumanPPI. ProtLigand (green) demonstrates better calibration than SaProt (blue) in both tasks. In the regression task, ProtLigand achieves lower ENCE, and in the classification task, it exhibits lower ECE while closely following the perfect line.

prediction *confidence* across most of its predictions. This confidence-driven distinction emphasizes the model's ability to make more decisive and biologically meaningful predictions.

As classification networks must not only be accurate but also reliable in their uncertainty estimates, calibrated confidence (Guo *et al.*, 2017) is essential for interpretability and downstream decision-making. We evaluate and plot the expected calibration error (ECE) (Guo *et al.*, 2017) for the HumanPPI task and the expected normalized calibration error (ENCE) (Levi *et al.*, 2019) for the Thermostability regression task, using 10 equal-width bins over the confidence or predicted value range, following the calibration equation (Guo *et al.*, 2017; Levi *et al.*, 2019). ProtLigand consistently exhibits improved calibration over SaProt, with lower ECE in classification (0.043 vs. 0.070) and reduced ENCE in regression (0.048 vs. 0.276). Reliability diagrams (see Supplementary Figure S2) show that ProtLigand tracks the identity line more closely than SaProt across the confidence bins.

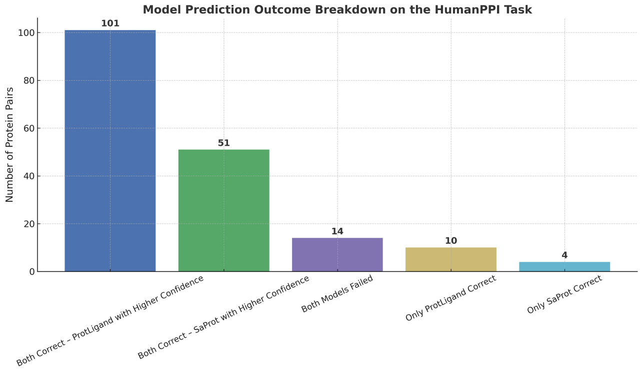

Fig. S3. Breakdown of the ProtLigand prediction outcomes on the HumanPPI test set on a representative run with a fixed random seed for reproducibility. Each bar represents the number of protein pairs grouped by correctness and confidence: whether both models were correct, failed, or only one model predicted correctly, further split by which model was more confident.

A key insight of our approach is that ligand information introduces additional functional context that complements traditional protein-level representations. Although prior models often capture structural and evolutionary features, the learned representations typically lack detailed information about how proteins interact with small molecules, information that is critical for many tasks, such as binding affinity prediction or drug response modeling.

Ligand signals encode important biochemical constraints, including charge distribution, hydrophobicity, and 3D conformation, all of which influence binding pocket compatibility. Incorporating these signals allows the model to better differentiate between proteins with similar sequences but divergent functions (e.g., paralogs) that cannot be distinguished through sequence alone. For properties like thermostability, ligand properties can have stabilizing or destabilizing effects on the protein–ligand complex. Including ligand features enables the model to reason about these interactions and capture their impact on protein function.

Overall, the ligand serves as a task-specific lens, enabling the model to refine its predictions based on real molecular interactions rather than relying solely on static structural features, resulting in higher confidence of the model on its predictions, leading to performance improvements as reflected in the results on the benchmarks.

S3 Ablation Tests

S3.1 Ligand Generator Ablation

To evaluate the importance of the ligand generator component in the ProtLigand architecture, we conduct an ablation experiment by replacing the generator with a random representation drawn from a normal distribution (corresponding in shape to the generated ligand representations).

Supplementary Table S5 presents the comparison across the six benchmark tasks. Replacing the generator leads to consistent drops in performance across all tasks, particularly in HumanPPI and Thermostability, where ligand context is known to play a key regulatory role. This highlights the contribution of the ligand generator in producing meaningful proxy representations, enabling ProtLigand to capture biochemically relevant signals that guide downstream prediction.

S3.2 Cross-Attention Ablation

To evaluate the contribution of the cross-attention in modeling the interaction between protein and ligand representations,

Table S5. Ablation study of the ligand generator. We compare the full model to a variant without the ligand generator, replaced by a random ligand representation. Results are reported as mean ± std over 3 independent fine-tuning runs.

Task	Full Model	Random Ligand
HumanPPI (Acc%)	90.00 ± 0.5	87.78 ± 0.46
Thermostability (ρ)	0.731 ± 0.02	0.716 ± 0.002
Metal Ion Binding (Acc%)	77.54 ± 0.18	74.89 ± 0.25
DeepLoc Binary (Acc%)	94.02 ± 0.21	93.38 ± 0.20
DeepLoc Sub. (Acc%)	83.88 ± 0.07	83.11 ± 0.28
EC ($F_{\max}$)	0.887 ± 0.002	0.878 ± 0.002

we conduct an ablation study in which the cross-attention layer is removed. Instead, we concatenate the protein and ligand representations and project them through a single linear layer to match the protein representation dimension. Specifically, we replace the attention block with: $\hat{z}_l = \text{Linear}\left(\llbracket z_p \parallel z'_l \rrbracket\right)$ where $\llbracket \cdot \parallel \cdot \rrbracket$ denotes vector concatenation, z'_l is the ligand representation and z_p is the protein representation (see Supplementary Section S1.0.1). This configuration removes explicit modeling of interaction patterns between the two modalities, relying instead on a fixed, shallow fusion.

Supplementary Table S6 presents the comparison across the six benchmark tasks. While the concatenation-based model performs reasonably well, it consistently underperforms the full cross-attention model across all tasks. This performance gap demonstrates that shallow fusion of protein and ligand representations is insufficient for capturing the nuanced, task-specific dependencies between modalities. Nevertheless, the concatenation-based model still outperforms the variant with a random ligand representation (Supplementary Table S5), indicating that even simpler integration of ligand features is beneficial for downstream performance. The constant improvements with cross-attention highlight its role in enriching the protein representations through more informative ligand-contextual interactions.

Table S6. Ablation study of the cross-attention mechanism. We compare the full model to a variant where protein and ligand representations are concatenated and linearly projected. Results are mean ± std over 3 independent fine-tuning runs.

Task	Full Model	Concatenation
HumanPPI (Acc%)	90.00 ± 0.5	88.33 ± 0.38
Thermostability (ρ)	0.731 ± 0.02	0.724 ± 0.004
Metal Ion Binding (Acc%)	77.54 ± 0.18	75.59 ± 0.19
DeepLoc Binary (Acc%)	94.02 ± 0.21	93.55 ± 0.23
DeepLoc Sub. (Acc%)	83.88 ± 0.07	83.33 ± 0.18
EC ($F_{\max}$)	0.887 ± 0.002	0.882 ± 0.002

S3.3 Robustness Across Sequence-Identity Bins

We evaluate ProtLigand's ability to generalize beyond its pre-training distribution by stratifying the HumanPPI test set based on sequence similarity to PDBbind, the corpus used during pre-training. Each HumanPPI test example consists of a protein pair; for each pair, we compute the Needleman–Wunsch sequence identity of both proteins to their respective closest match in the pre-training set. We then average the two identities to obtain a single similarity score for the pair. The full test set is sorted by these pairwise similarity scores and partitioned into five equal-sized bins.

Supplementary Figure S4 plots the average HumanPPI classification accuracy for each bin against its mean sequence identity. As expected, accuracy increases with similarity, but performance remains strong even in the low-similarity regime: in the lowest bin, where mean identity is just 16.02%, accuracy reaches 83.3%; in the highest bin, where identity approaches 28.5%, performance climbs to 94.4%. These results confirm that ProtLigand generalizes beyond memorization of homologous sequences. It maintains robust performance even when both proteins in a test pair are dissimilar to any pre-training example, highlighting its potential to operate effectively in real-world contexts such as metabolite-rich or novel ligand datasets, where target proteins may lie well outside the PDBbind manifold.

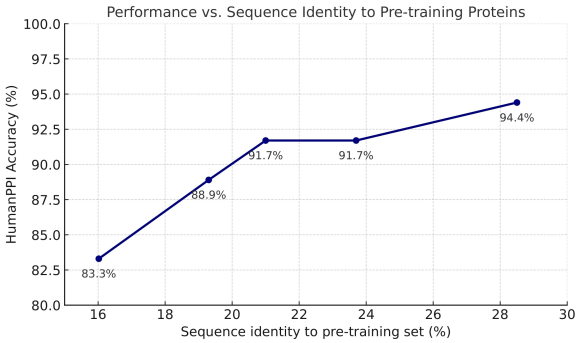

Fig. S4. ProtLigand performance on the HumanPPI task as a function of pairwise sequence identity to proteins in the pre-training set. For each test pair, we compute the mean identity of the two proteins to their closest pre-training counterparts. The test set is split into five equal-sized bins by this score. Each point shows the average classification accuracy within a bin, plotted against that bin's mean identity.

S3.4 Robustness to Low-Confidence Regions

Protein structures derived from AlphaFold2 can contain low-confidence regions, which are reflected in segments with low pLDDT scores. These regions lack reliability for practical use. To assess the robustness of ProtLigand in these scenarios, we simulate low-confidence regions by randomly masking a percentage of structure tokens with the special token "#", following our filtering strategy. We then fine-tune the model on the DeepLoc dataset (Armenteros, 2017) and evaluate performance across varying mask rates. Figure S5 presents the accuracy of ProtLigand under different mask rates, ranging from 0.0 to 1.0, compared to ESM-2 (Lin *et al.*, 2023), a top-performing sequence-only PLM. As expected, as the mask rate increases, performance gradually degrades due to the essential information loss. However, ProtLigand remains competitive with ESM-2, even when all structure tokens are masked. This indicates that, while structural information boosts performance, the model still retains meaningful representations from sequence or partial structure input due to low-confidence regions.

Table S7. Benchmark dataset statistics for the six benchmark tasks.

Dataset	Category	Evaluation Metric	Train	Valid	Test
HumanPPI (Xu *et al.*, 2022)	PPI Prediction	Accuracy	26319	234	180
Thermostability (Dallago *et al.*, 2021)	Protein Function Prediction	Spearman's ρ	5056	639	1336
Metal Ion Binding (Hu *et al.*, 2022)	Protein Function Prediction	Accuracy	5067	662	665
DeepLoc (Binary) (Armenteros, 2017)	Protein Localization Prediction	Accuracy	5477	1336	1731
DeepLoc (Subcellular) (Armenteros, 2017)	Protein Localization Prediction	Accuracy	8747	2191	2747
EC (Gligorijević *et al.*, 2021)	Protein Annotation Prediction	$F_{\max}$	13089	1465	1604

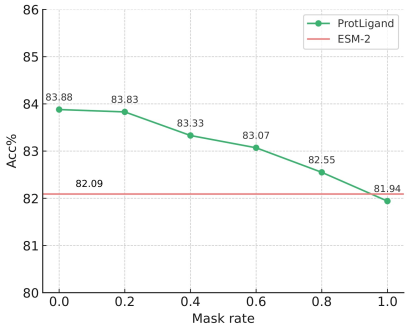

(a) DeepLoc (Subcellular)

Fig. S5. ProtLigand performance on the DeepLoc task as a function of various mask rates applied to structure tokens, compared to ESM-2 performance.

S3.5 Effect of AlphaFold2 Recycling Steps

AlphaFold2 refines its structure predictions through an internal recycling mechanism, where intermediate representations are iteratively updated. The default number of recycling steps is 3, but it has been hypothesized that increasing this number could enhance downstream performance. In Supplementary Figure S6, we report the performance of ProtLigand on the DeepLoc (subcellular) task using AlphaFold2 structures generated with 3, 6, and 12 recycling steps. We observe that increasing the number of recycles does not lead to a statistically significant improvement in classification accuracy. This suggests that while additional recycling may improve fine-grained structural quality, it provides minimal benefit for ProtLigand in this task. These findings are consistent with other downstream tasks and previous reports in the literature (Adiyaman *et al.*, 2023), which show that enhanced structural fidelity from deeper recycling does not necessarily translate to improved performance in downstream predictive models.

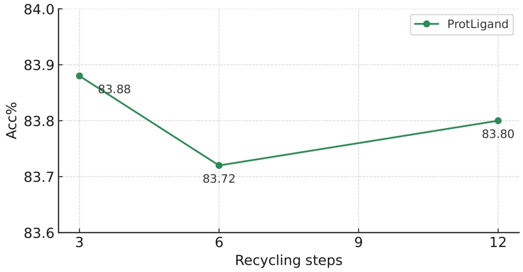

(a) DeepLoc (Subcellular)

Fig. S6. ProtLigand performance on the DeepLoc task as a function of AlphaFold2 Recycling Steps.

S4 Datasets Overview

S4.1 Benchmark datasets

Supplementary Table S7 summarizes the benchmark datasets, including task categories, evaluation metrics, and the sizes of training, validation, and test splits.

S4.2 Pre-training Dataset

S4.2.1 Proteins Overview

We provide in the Supplementary Table S8 an overview of the distribution of the protein-ligand complex by protein type in our pre-training dataset.

To assess structural diversity at the fold level, we mapped all unique proteins in the pre-training set to their corresponding CATH superfamilies (Orengo *et al.*, 1997). Each protein may contain multiple domains, and each domain was assigned to a CATH superfamily. Table S9 reports the total number of superfamily-level domain assignments. Since many superfamilies occur only a few times, we list the most frequent folds explicitly and group all others under Other.

Table S8. Total number of protein–ligand complexes for each protein type in the pre-training dataset.

Protein Type	Protein Amount
Transferase	4941
Hydrolase	4843
Transcription	960
Lyase	848
Transport	589
Oxidoreductase	562
Ligase	434
Isomerase	320
Chaperone	297
Membrane	270
Metal-containing	112
Viral	18
Other	3199

Reliability of AlphaFold2 predictions can be challenging in specific cases, such as for metamorphic proteins, which AlphaFold2 has been shown to mispredict (Chakravarty and Porter, 2022). However, in our pre-training dataset, only approximately 0.2–0.5% of proteins can plausibly be classified as metamorphic (fold-switching) proteins, a rate consistent with prior findings in the PDB dataset (Porter and Looger, 2018). Thus, for most modeling tasks, the dataset can be effectively treated as metamorphic-free.

In addition, we observe that only 10 proteins, which are approximately 0.29% of all proteins in the dataset, are partner proteins, such as SUMO1 (UniProt ID: P63165). Therefore, this

Table S9. Total number of domain assignments per CATH superfamily in the pre-training dataset.

CATH Superfamily	Domain Count
Transferase domain	227
Phosphorylase Kinase	204
Immunoglobulin-like	105
P-loop NTP hydrolase	95
Zinc finger (C3HC4)	65
PH / PTB domain	59
SH3 domain	56
PI3K catalytic subunit	53
Aldolase class I	50
Glycosidase	44
Rossmann fold	43
SH2 domain	42
Other	3911

fraction is too small to have a meaningful impact on model training or evaluation.

In our dataset, the average length of protein sequences is 800.28 amino acids, with a standard deviation of 250.28, reflecting the broad diversity of full-length proteins and their interaction contexts.

S4.2.2 Ligands Overview

To better understand the chemical diversity of ligand interactions, we classified each ligand into broad functional categories using the RDKit cheminformatics library, based on their molecular SMILES representations. As shown in Supplementary Table S10, the dataset spans a broad range of ligand types, including lipids, drug-like molecules, peptides, and nucleotides, reflecting the functional diversity of biologically relevant binding events.

In addition, 21.7% of the ligands contain at least one bound metal ion, with Zn^{2+} (6.2%), Mg^{2+} (5.0%), Ca^{2+} (4.2%), Mn^{2+} (1.4%), and Fe^{2+}/Fe^{3+} (1.0%) being the most prevalent, highlighting the importance of metal-mediated interactions across the dataset.

Table S10. Distribution of ligand types in the pre-training dataset.

Ligand Type	Count
Lipid	6970
Drug-like	4526
Peptide	2358
Carbohydrate	1972
Nucleotide	1500
Metal-containing	35
Other	32

References

Adiyaman, R., Edmunds, N. S., Genc, A. G., Alharbi, S. M. A., and McGuffin, L. J. (2023). Improvement of protein tertiary and quaternary structure predictions using the refold refinement method and the alphafold2 recycling process. *Bioinformatics Advances*, **3**(1), vbad078.

Armenteros, A. (2017). Deeploc: prediction of protein subcellular localization using deep learning. *Bioinformatics*, **33**, 3387–3395.

Capel, H. L., Weiler, R., Dijkstra, M. J. J., Vleugels, R., Bloem, P., and Feenstra, K. A. (2022). Proteinglue multi-task benchmark suite for self-supervised protein modeling. *Scientific Reports*, **12**.

Chakravarty, D. and Porter, L. L. (2022). Alphafold2 fails to predict protein fold switching. *Protein Science*, **31**(6), e4353.

Chithrananda, S., Grand, G., and Ramsundar, B. (2020). Chemberta: Large-scale self-supervised pretraining for molecular property prediction. *ArXiv*, **abs/2010.09885**.

Dallago, C., Mou, J., Johnston, K. E., Wittmann, B., Bhattacharya, N., Goldman, S., Madani, A., and Yang, K. K. (2021). FLIP: Benchmark tasks in fitness landscape inference for proteins. In *Thirty-fifth Conference on Neural Information Processing Systems Datasets and Benchmarks Track (Round 2)*.

Devlin, J., Chang, M.-W., Lee, K., and Toutanova, K. (2019). Bert: Pre-training of deep bidirectional transformers for language understanding. In *North American Chapter of the Association for Computational Linguistics*.

Gligorijević, V., Renfrew, P. D., Kosciólek, T., Leman, J. K., Berenberg, D., Vatanen, T., Chandler, C., Taylor, B. C., Fisk, I., Vlamakis, H., Xavier, R. J., Knight, R., Cho, K., and Bonneau, R. (2021). Structure-based protein function prediction using graph convolutional networks. *Nature Communications*, **12**.

Guo, C., Pleiss, G., Sun, Y., and Weinberger, K. Q. (2017). On calibration of modern neural networks. In *Proceedings of the 34th International Conference on Machine Learning - Volume 70*, ICML'17, page 1321–1330. JMLR.org.

Hu, M., Yuan, F., Yang, K. K., Ju, F., Su, J., Wang, H., Yang, F., and Ding, Q. (2022). Exploring evolution-aware & -free protein language models as protein function predictors. In *Neural Information Processing Systems*.

Humphreys, I. R., Pei, J., Baek, M., Krishnakumar, A., Anishchenko, I., Ovchinnikov, S., Zhang, J., Ness, T. J., Banjade, S., Bagde, S. R., Stancheva, V. G., Li, X.-H., Liu, K., Zheng, Z., Barrero, D. J., Roy, U., Kuper, J., Fernández, I. S., Szakal, B., Branzei, D., Rizo, J., Kisker, C., Greene, E. C., Biggins, S., Keeney, S., Miller, E. A., Fromme, J. C., Hendrickson, T. L., Cong, Q., and Baker, D. (2021). Computed structures of core eukaryotic protein complexes. *Science*, **374**(6573), eabm4805.

Levi, D., Gispan, L., Giladi, N., and Fetaya, E. (2019). Evaluating and calibrating uncertainty prediction in regression tasks. *Sensors (Basel, Switzerland)*, **22**.

Lin, Z., Akin, H., Rao, R., Hie, B., Zhu, Z., Lu, W., Smetanin, N., Verkuil, R., Kabeli, O., Shmueli, Y., dos Santos Costa, A., Fazel-Zarandi, M., Sercu, T., Candido, S., and Rives, A. (2023). Evolutionary-scale prediction of atomic-level protein structure with a language model. *Science*, **379**(6637), 1123–1130.

Orengo, C. A., Michie, A. D., Jones, S., Jones, D. C., Swindells, M. B., and Thornton, J. M. (1997). Cath–a hierarchic classification of protein domain structures. *Structure*, **5 8**, 1093–108.

Porter, L. L. and Looger, L. L. (2018). Extant fold-switching proteins are widespread. *Proceedings of the National Academy of Sciences*, **115**(23), 5968–5973.

Rives, A., Goyal, S., Meier, J., Guo, D., Ott, M., Zitnick, C. L., Ma, J., and Fergus, R. (2019). Biological structure and function emerge from scaling unsupervised learning to 250 million protein sequences. *Proceedings of the National Academy of Sciences of the United States of America*, **118**.

Shin, J.-E., Riesselman, A. J., Kollasch, A. W., McMahon, C., Simon, E., Sander, C., Manglik, A., Kruse, A. C., and Marks, D. S. (2021). Protein design and variant prediction using autoregressive generative models. *Nature Communications*, **12**.

Su, J., Han, C., Zhou, Y., Shan, J., Zhou, X., and Yuan, F. (2024). Saprot: Protein language modeling with structure-aware vocabulary. In *The Twelfth International Conference on Learning Representations*.

van Kempen, M., Kim, S., Tumescheit, C., Mirdita, M., Lee, J., Gilchrist, C. L., Söding, J., and Steinegger, M. (2022). Fast and accurate protein structure search with foldseek. *Nature Biotechnology*, **42**, 243 – 246.

Vaswani, A., Shazeer, N. M., Parmar, N., Uszkoreit, J., Jones, L., Gomez, A. N., Kaiser, L., and Polosukhin, I. (2017). Attention is all you need. In *Neural Information Processing Systems*.

Xu, M., Zhang, Z., Lu, J., Zhu, Z., Zhang, Y., Ma, C., Liu, R., and Tang, J. (2022). Peer: A comprehensive and multi-task benchmark for protein sequence understanding. In *Thirty-sixth Conference on Neural Information Processing Systems Datasets and Benchmarks Track*.

Yang, K. K., Zanichelli, N., and Yeh, H. (2022). Masked inverse folding with sequence transfer for protein representation learning. *Protein Engineering, Design and Selection*, **36**, gzad015.

Zhang, Z., Xu, M., Jamasb, A. R., Chenthamarakshan, V., Lozano, A., Das, P., and Tang, J. (2023a). Protein representation learning by geometric structure pretraining. In *The Eleventh International Conference on Learning Representations*.

Zhang, Z., Wang, C., Xu, M., Chenthamarakshan, V., Lozano, A. C., Das, P., and Tang, J. (2023b). A systematic study of joint representation learning on protein sequences and structures.
